# Understanding Clinical and Biophysical Differences Between Non‐Cross‐Linked and Cross‐Linked Hyaluronic Acid Dermal Fillers

**DOI:** 10.1111/jocd.70595

**Published:** 2025-12-24

**Authors:** Silvia Fontenete, Carlos Bravo, Michael Alfertshofer, Sebastian Cotofana

**Affiliations:** ^1^ Medical Department BioScience GmbH Madrid Spain; ^2^ Private Practice San José Costa Rica; ^3^ Department of Oromaxillofacial Surgery Ludwig‐Maximilians‐University Munich Munich Germany; ^4^ Department of Plastic Surgery Vanderbilt University Medical Center Nashville Tennessee USA; ^5^ Department of Dermatology Erasmus Medical Centre Rotterdam the Netherlands; ^6^ Centre for Cutaneous Research, Blizard Institute Queen Mary University of London London UK; ^7^ Department of Plastic and Reconstructive Surgery, Guangdong Second Provincial General Hospital Guangzhou Guangdong Province China

**Keywords:** cross‐linking, hyaluronic acid, non‐Newtonian fluids, rheology, soft tissue dermal filler

## Abstract

**Background:**

The rising popularity of injectable soft‐tissue fillers in esthetic medicine is driven by their potential to provide facial rejuvenation through minimally invasive techniques. Hyaluronic acid (HA)‐based fillers exhibit distinct biophysical characteristics, including rheological properties which are critical for their behavior under mechanical stress. This study aims to compare the viscoelastic properties of commercially available crosslinked (CFs) and non‐crosslinked HA fillers (NCFs).

**Methods:**

A total of 28 commercially available HA fillers, including both non‐cross‐linked (*n* = 3) and cross‐linked types (*n* = 25), were investigated for their rheological properties. Rheological parameters such as storage modulus (*G′*), loss modulus (*G″*), loss tangent (*tan δ*), and complex modulus (G*) were measured over a frequency range of 0.1–10 Hz.

**Results:**

NCFs showed greater variation in G′ with a mean of 3263% [range: 1767–4177] compared to crosslinked fillers with 247.60% [range: 85–720]. The calculated difference between the change in percent of CFs versus NCFs was 3016% (755) for G′ with *p* < 0.001, whereas it was 926% (498) for G″ with *p* < 0.001, and it was −154% (25.8) for tan‐delta with *p* < 0.001, and 966% (147) for G* with *p* < 0.001.

**Conclusions:**

NCFs and CFs exhibit distinct rheological profiles, with NCFs demonstrating a greater change in their initial rheologic properties when exposed to mechanical stress. This specific biophysical behavior (increase in stiffness and viscosity) predisposes NCFs for dermal structural support with the respective subdermal applications, whereas CFs seem to be more suitable for deep soft tissue injections, offering volumization.

## 1 Introduction

In recent years, the popularity of injectable soft tissue fillers in aesthetic medicine has surged [1] due to their ability to provide effective facial rejuvenation and aesthetic improvement through minimally invasive injection techniques [2].

Hyaluronic acid (HA)‐based soft‐tissue fillers exhibit distinct biophysical characteristics. These include variations in rheological properties and cohesivity levels, which influence their behavior and effects on facial and non‐facial soft tissues [3, 4, 5]. Among these biophysical characteristics, rheological parameters are particularly important, as they describe how a filler responds to mechanical forces during and after injection [4, 6]. Depending on the filler's biophysical characteristics and material composition, the injected product can exhibit both solid‐like (i.e., elastic) and liquid‐like (i.e., viscous) behavior. Once in the body, these gels are subjected to shear stress, compression, and stretching forces within soft tissues [4, 6].

In a previous study, Cotofana and colleagues analyzed the viscoelastic properties of 35 randomly selected HA‐based fillers by measuring their rheologic characteristics at various angular frequencies ranging from 0.1 to 100 radian/second [7]. The results of that study revealed an increase in the initial elastic modulus (G′) when the angular frequency was increased suggesting a transition from softer to harder fillers if more shear forces affect the filler materials. Additionally, the authors observed fluctuations in the viscous modulus (G″), indicating changes in the fluidity of the fillers. The observed variations in rheological properties could play an important role in determining the safety profile and the esthetic outcome as the face is composed of high and low mobility regions which can affect the implanted materials in various ways [7].

However, in their study, Cotofana et al. [7] investigated the rheologic properties of cross‐linked HA‐based soft tissue fillers (CFs) only and did not include non‐cross‐linked HA‐based materials (NCFs). NCFs, also known as skin boosters, are primarily used for supporting dermal integrity through subdermal injections and have different treatment and aesthetic profiles [8, 9, 10]. Understanding this profile is crucial for treatment success and managing patients expectations especially when it comes to addressing volume loss and dermal rhytids.

However, the general understanding of the rheologic profile of NCFs is widely missing leaving the esthetic community with a paucity of reliable information about NCFs. Therefore, it is the aim of this study to describe the differences in rheological properties between CFs and NCFs and to specify their performance and suitability for various esthetic clinical applications.

## 2 Material and Methods

### 2.1 Study Sample

A total of 28 randomly selected HA‐based soft tissue fillers from different manufacturing technologies were included in this study (Table 1). The products assessed in the study were purchased specifically for the purposes of this study of which *n* = 3 were NCFs and *n* = 25 were CFs. All products were sent to an independent laboratory (Aptol, Copenhagen, Denmark) for rheological testing to assure data independence and result validity. This study was carried out between January 2024 and July 2024.

**TABLE 1:** Overview of the 28 randomly selected HA‐based soft tissue fillers from different manufacturing technologies analyzed in this study.

Brand	Product commercial name	Assigned experimental code	Cross‐linking agent and concentration	Cross‐linked HA concentration [mg/mL]	Non‐cross‐linked HA concentration [mg/mL]	Lidocaine [mg/mL]	Additional additives	Recommended area of use
BioScience GMBH	Hyacorp fine	BS1	NA	NA	14 mg/mL	NA	NA	Face, neck, décolleté, back of the hands
	Hyacorp Feel	BS2	BDDE (< 1 ppm)	18 mg/mL	2 mg/mL	NA	NA	Volume replacement (filling of folds), superficial folds, periorbital lines, perioral lines
	Hyacorp lips	BS3	BDDE (< 1 ppm)	16 mg/mL	2 mg/mL	NA	NA	Volume and contour of the lips
	Hyacorp face	BS4	BDDE (< 1 ppm)	20 mg/mL	2 mg/mL	NA	NA	Restoration of the facial volume and contour replaces lost HA in the skin is used for volume replacement (filling of folds), medium to deep folds, nasolabial folds, cheek area, glabella folds
	Genefill fine	BS5	NA	NA	14 mg/mL	NA	NA	Face, neck, décolleté, back of the hands
	Genefill soft touch	BS6	BDDE (< 1 ppm)	16 mg/mL	2 mg/mL	NA	NA	Volume replacement (filling of folds), superficial folds, periorbital lines, perioral lines
	Genefill soft fill	BS7	BDDE (< 1 ppm)	20 mg/mL	2 mg/mL	NA	NA	Volume replacement (filling folds), medium to deep folds, nasolabial folds, cheek area, lip augmentation, glabella folds
Allergan	Juvederm volbella	C1	BDDE (< 2 ppm)	15 mg/mL	NA	0.3%	NA	Lip augmentation and correction of perioral lines and infraorbital
	Juvederm ultra 2	C2	BDDE (< 2 ppm)	24 mg/mL	NA	0.3%	NA	Moderate to severe facial wrinkles and folds
	Juvederm ultra 3	C3	BDDE (< 2 ppm)	24 mg/mL	NA	0.3%	NA	Perioral area for lip augmentation
	Juvederm volux	C4	BDDE (< 2 ppm)	25 mg/mL	NA	0.3%	NA	Jawline definition
Merz	Belotero ballance	C5	BDDE (0.28 mg/mL)	22.5 mg/mL	NA	0.3%	NA	Correction of moderate lines: Nasolabial folds (nose to mouth lines) Moderate perioral lines (smoker's lines) Lip contouring; marionette lines
	Belotero lips shape	C6	BDDE (0.28 mg/mL)	22.5 mg/mL	NA	0.3%	NA	Lips
	Belotero intense	C7	BDDE (0.28 mg/mL)	25.5 mg/mL	NA	0.3%	NA	Correction of deep lines: deep nasolabial folds (nose to mouth lines) Lip volumes, Marionette lines. Filling deep wrinkles and folds, restoring and enhancing the volume of soft tissues, and correcting facial scars.
	Belotero Volume	C8	BDDE (0.28 mg/mL)	26 mg/mL	NA	0.3%	NA	Restoration of facial volume in cheeks and cheekbones, chin, facial volume loss, temples, and jawline.
Galderma	Restylane defyne	C9	BDDE (< 1 ppm)	20 mg/mL		0.3%	NA	Correction of moderate to severe deep facial wrinkles and folds, such as nasolabial folds and augmentation of the chin.
	Restylane kysse	C10	BDDE (< 1 ppm)	20 mg/mL		0.3%	NA	Lips
Teoxane	Teosyal rha 1	C11	BDDE (< 1 ppm)	15 mg/mL		0.3%	NA	Fine lines on the face, such as the perioral rhytids, on the neck and on the décolleté
	Teosyal rha 3	C12	BDDE (< 1 ppm)	23 mg/mL	NA	0.3%	NA	Moderate to severe folds, such as nasolabial folds and marionette lines, and for lip plumping
	Teosyal rha kiss	C13	BDDE (< 1 ppm)	25 mg/mL	NA	0.3%	NA	Lips
	Teosyal rha 4	C14	BDDE (< 1 ppm)	23 mg/mL	NA	0.3%	NA	Contouring and reshaping of areas such as the temples, cheeks, jawlines, and chin
Vivacy	Stylage hydromax	C15	BDDE (< 2 ppm)	12.5 mg/g	NA	NA	IPN‐like + sorbitol	Skin moisturization and elasticity
	Stylage S	C16	BDDE (< 2 ppm)	16 mg/g	NA	NA	IPN‐like + sorbitol	Fine to medium wrinkles, perioral area, and hydration
	Stylage special lips	C17	BDDE (< 2 ppm)	18.5 mg/g	NA	NA	IPN‐like + sorbitol	Lip augmentation for light volume, definition, and hydration
	Stylage L	C18	BDDE (< 2 ppm)	24 mg/g	NA	NA	IPN‐like + sorbitol	Deep to very deep wrinkles

### 2.2 Rheology Measurements

For in vitro measurements, the following materials were used: Dulbecco's phosphate buffered saline (PBS) without calcium and magnesium (No. 17‐51GF, Lonza Group, Basel, Switzerland), distilled water (Walter Schmidt Chemie GmbH, Berlin, Germany), 10 mL centrifuge tubes (TT19.1, Carl Roth, Karlsruhe, Germany), and a connector (PZN 0 545 0245, B. Braun Melsungen AG, Melsungen, Germany).

The testing employed an oscillatory method to investigate the rheological properties of the samples when subjected to deformations across a range of frequencies. Rheological parameters were measured using a Discovery Hybrid Rheometer (DHR) with a controlled stress single head, employing a 60mm parallel plate, Peltier plate aluminum at 25°C, with a 2% strain. Measurements were standardized and controlled for temperature and relative humidity. The average room temperature for all measurements was maintained at 21.0°C ± 1.5°C, and the average relative humidity was 24.0% ± 5.0%. Frequencies between 0.1 and 10 Hz were used to determine the rheological parameters: G′, G″, and tan δ (loss tangent). The elastic or storage modulus (G′) quantifies the gel's stiffness and its ability to resist deformation, which is essential for shape retention under compression or muscle movement and quantifies the ability of each material to recover its original shape following the application of shear forces. The viscous or loss modulus (G″) measures the gel's ability to dissipate energy during shear stress, reflecting its fluidity and assesses the material's propensity to exhibit liquid‐like flow behavior. The loss tangent, tan δ (G″/G′), provides insight into the balance between elasticity and fluidity—a high value indicates a softer, more flowing filler, while a low value reflects firmer, more resilient gels. This ratio, provides insight into the relative balance between viscous and elastic responses within each filler. Finally, the complex modulus (G)* expresses the total resistance of the gel to deformation, combining both elastic (recoverable) and viscous (non‐recoverable) responses [4, 5, 6].

The complex modulus, was calculated as follows: $G^{*}=\sqrt{{\left(G^{\prime }\right)}^{2}+{\left(G^{\prime \prime }\right)}^{2}}$.

The comprehensive viscoelastic properties of a sample, integrating both G′ and G″, are encapsulated by the complex modulus, G* [4, 5, 6].

### 2.3 Statistical Analysis

Descriptive and comparative analyses were calculated using SPSS version 27 (IBM, Armonk, NY, USA). A total of 3 NCFs were compared to a total of 25 CFs which resulted in the use of non‐parametric testing (Mann–Whitney *U*‐test) alongside the assumption of non‐equal variances. Results are presented as mean values paired with the 1× standard deviation (mean (SD)) whereas differences are provided as mean value and the respective standard error. Results were considered statistically significant at a probability level of *p* < 0.05 to guide conclusions.

### 2.4 Ethics Statement

As this study involved only in vitro analysis of commercially available products without any human or animal participants, formal ethical approval was not required.

## 3 Results

### 3.1 Non‐Cross‐Linked Fillers

**FIGURE 1:**
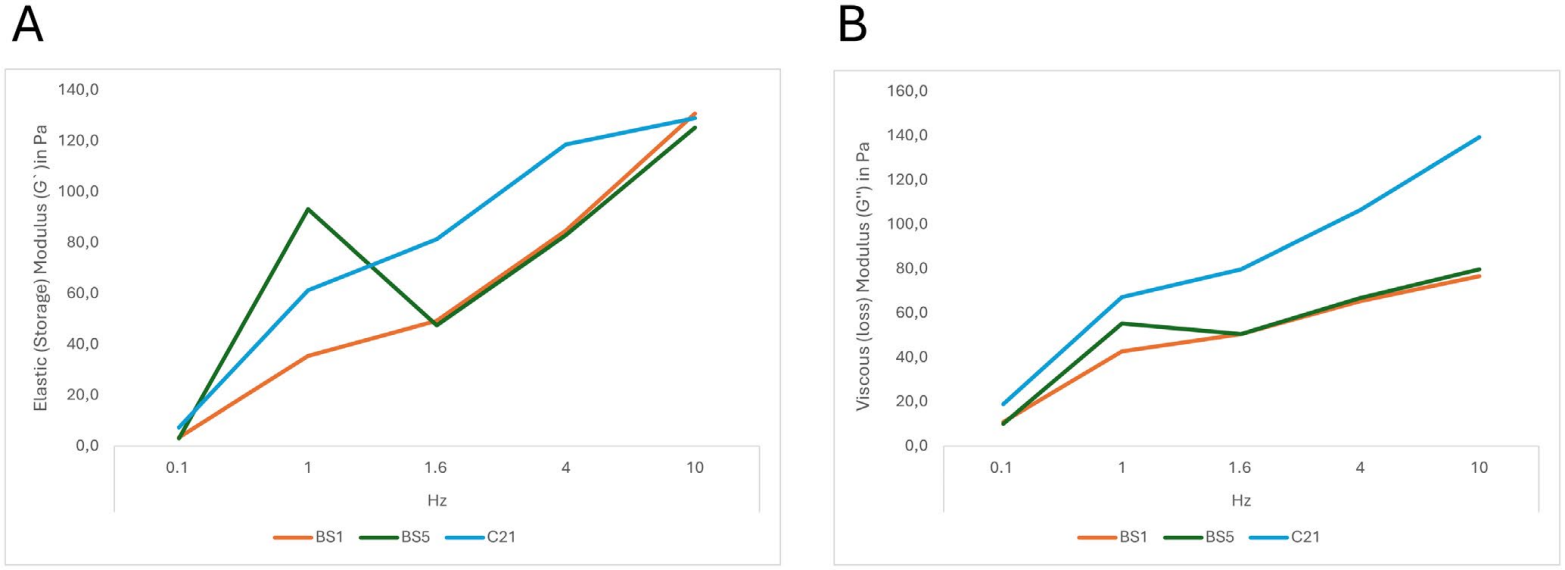
Line graph showing the elastic storage modulus (*G′*) and viscous loss modulus (*G″*) in pascals for tested non‐cross‐linked products (color‐encoded) at 0.1, 1, 1.6, and 10 Hz. (A) Elastic storage modulus (*G′*). (B) Viscous loss modulus (*G″*).

A total of three NCFs were investigated in this study with a mean G′ value of 4.57 (2.4) Pa, 63.33 (28.9) Pa, 59.10 (19.3) Pa, 95.50 (20.1) Pa, and 128.37 (2.8) Pa tested at 0.1, 1.0, 1.6, 4, and 10 Hz, respectively. The calculated average change in G′ between 0.1 and 10 Hz was 123.80 (3.1) Pa [range: 121.7 – 127.4] representing an increase of 3263% [range: 1767–4177] when compared to the values at 0.1 Hz. The respective increase in G″ was 1260% [range: 737–2253], whereas the increase in tan δ was 26.19% [range: 18–42], and in G* the increase was 1218% [range: 935–1401]. For more detailed data presentation see Table 2 and Figure 1.

**TABLE 2:** *G′*, *G″*, G* and tan *δ* values (in pascals) for the three measured non‐cross‐linked soft tissue fillers at 0.1, 1.0, 1.6, 4, and 10 Hz. Values represent mean values for duplicate measurements.

	Mean *G′* (Hz)						Mean *G″* (Hz)						Mean tan *δ* (Hz)						Mean G* (Hz)					
ID	0.1 Hz	1 Hz	1.6 Hz	4 Hz	10 Hz	Change (%)	0.1 Hz	1 Hz	1.6 Hz	4 Hz	10 Hz	Change (%)	0.1 Hz	1 Hz	1.6 Hz	4 Hz	10 Hz	Change (%)	0.1 Hz	1 Hz	1.6 Hz	4 Hz	10 Hz	Change (%)
BS1	3.4	35.5	49.3	84.8	130.8	3747.0	10.9	42.7	50.6	65.3	76.6	602.8	3.2	1.2	1.0	0.8	0.6	−81.8	11.5	55.5	70.6	107.0	151.5	1217.4
BS5	3.0	93.2	47.4	83.0	125.3	4076.7	10.1	55.4	50.6	66.8	79.7	689.1	3.4	0.2	1.1	0.8	0.6	−81.2	10.6	108	69.4	106.6	148.5	1300.9
C21	7.3	61.3	81.3	118.7	129.0	1667.1	18.9	67.3	79.8	106.4	139.3	637.0	2.6	1.1	1.0	0.9	1.1	−57.8	20.3	91.0	113.9	189.8	189.8	835.0

### 3.2 Cross‐Linked Fillers (CFs)

**FIGURE 2:**
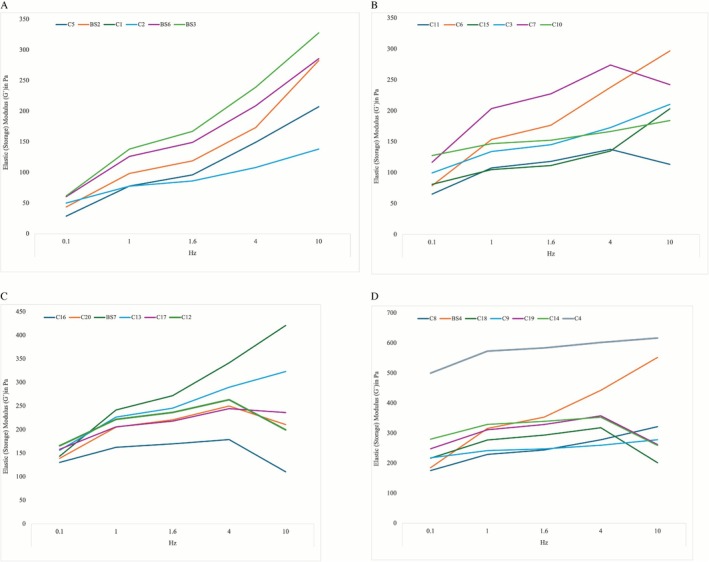
Line graph showing the elastic storage modulus (*G′*) in pascals for tested products (color‐encoded) at 0.1, 1, 1.6, and 10 Hz. (A) Products with a *G′* of less than 62 Pa at 0.1 Hz. (B) Products with a *G′* between 62 and 130 Pa at 0.1 Hz. (C) Products with a *G′* between 130 and 170 Pa at 0.1 Hz. (D) Products with a *G′* higher than 170 Pa at 0.1 Hz.

**FIGURE 3:**
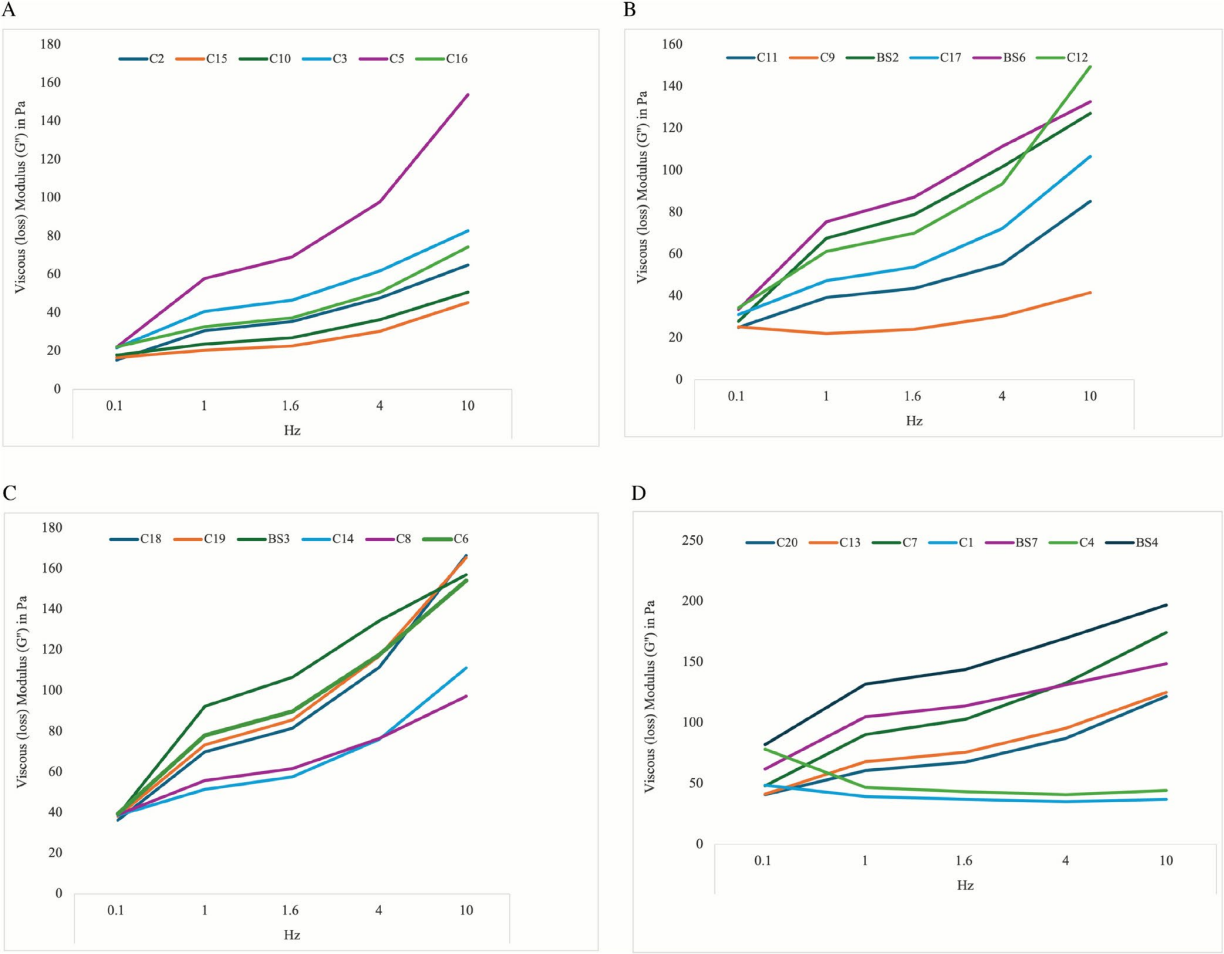
Line graph showing the viscous loss modulus (*G″*) in pascals for depicted, color‐encoded products at 0.1, 1, 1.6, and 10 Hz. (A) Products with a *G″* of less than 30 Pa at 0.1 Hz are depicted in this figure. (B) Products with a *G″* between 25 and 35 Pa at 0.1 Hz are represented in this figure. (C) Products with a *G″* between 35 and 40 Pa at 0.1 Hz. (D) Products with a *G″* higher than 40 Pa at 0.1 Hz.

**FIGURE 4:**
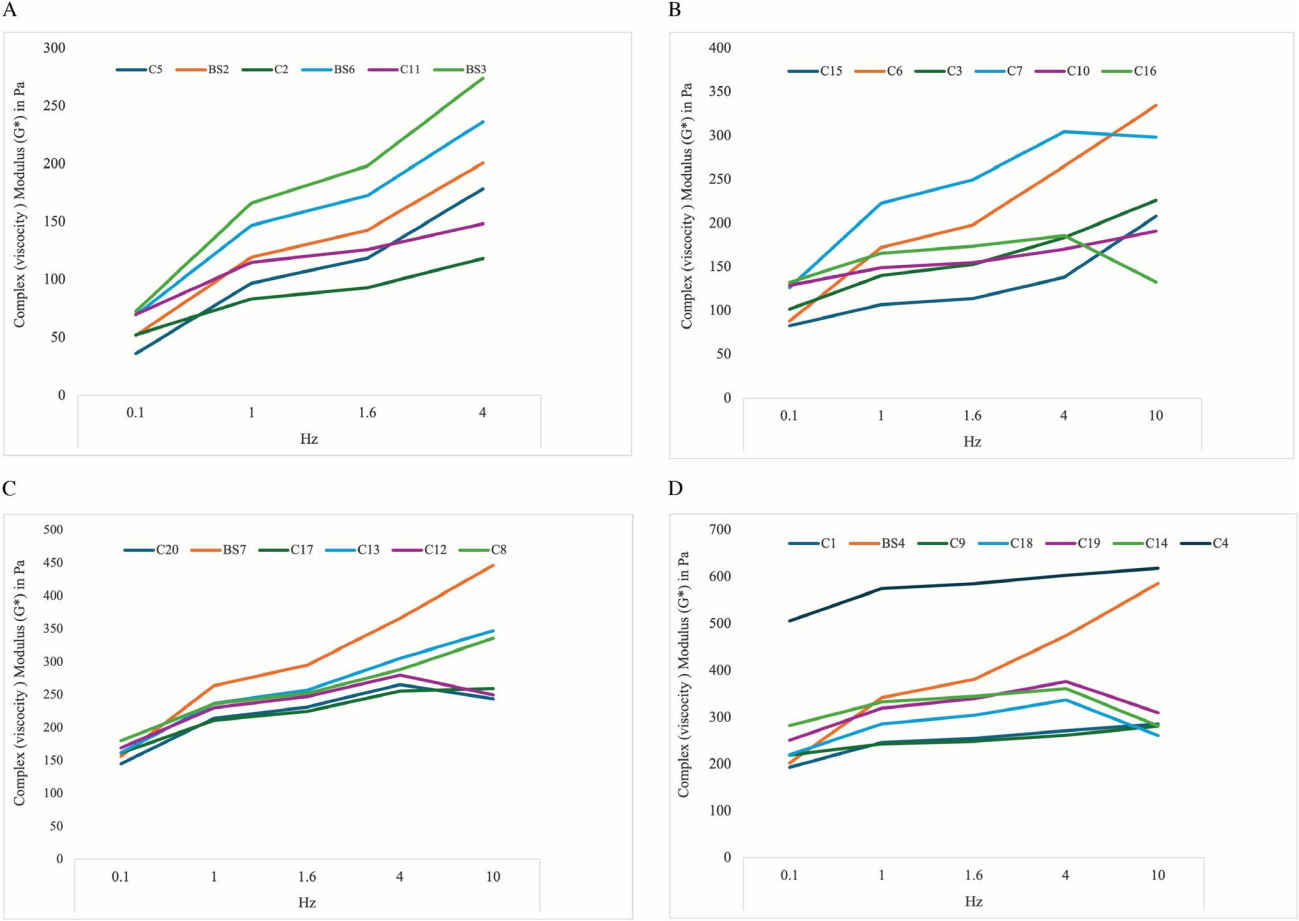
Line graph showing the complex viscosity modulus (G*) in pascals for depicted, color‐encoded products at 0.1, 1, 1.6, and 10 Hz. (A) Products with a G* of less than 75 Pa at 0.1 Hz are depicted. (B) Products with a G* between 75 and 135 Pa is represented. (C) Products with a G* between 135 and 180 Pa is represented. (D) Products with a G* higher than 180 Pa is represented.

**FIGURE 5:**
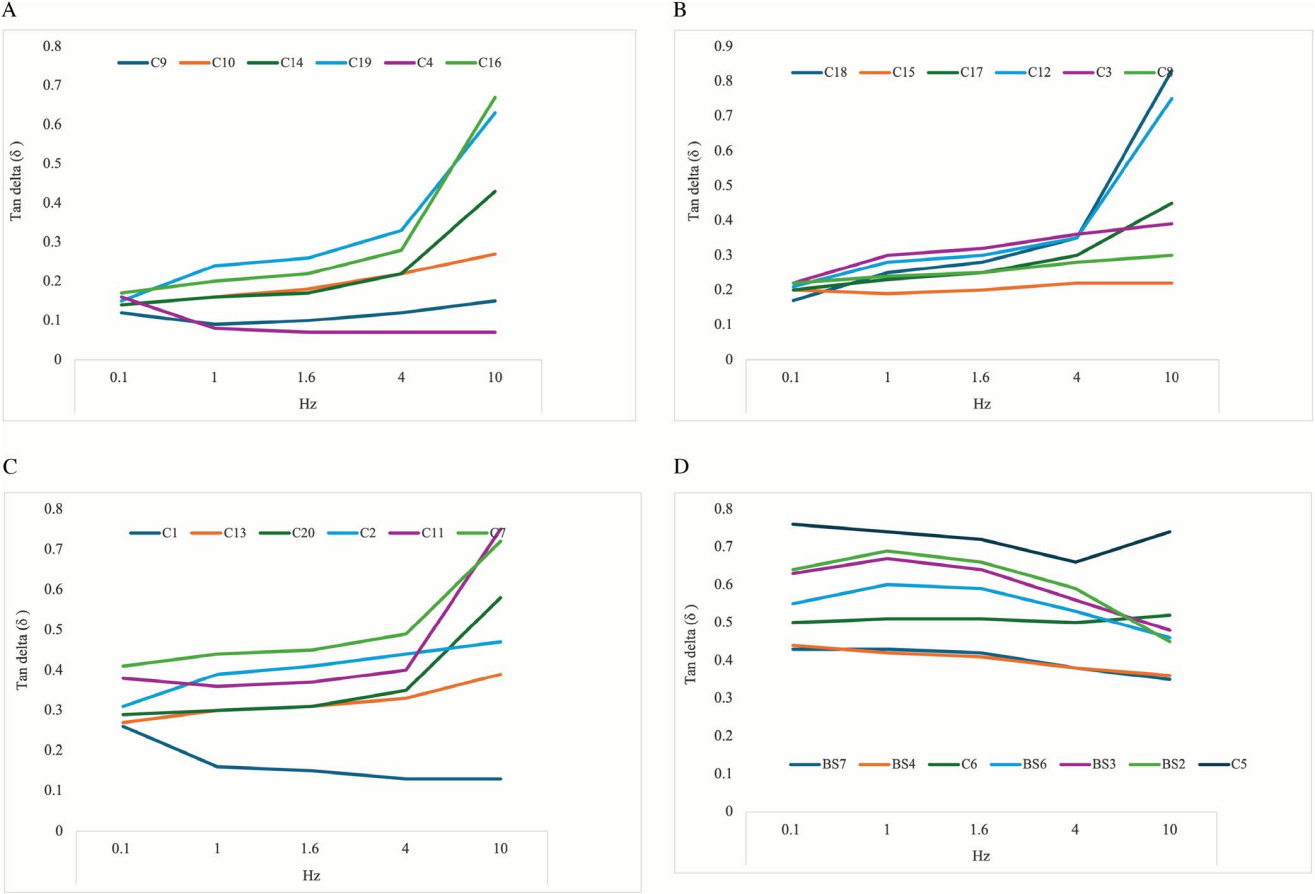
Line graph showing the tan *δ* for depicted, color‐encoded products at 0.1, 1, 1.6, and 10 Hz. (A) Products with a tan *δ* of less than 0.17 at 0.1 Hz are depicted. (B) Products with a tan *δ* between 0.17 and 0.22 at 0.1 Hz are represented. (C) Products with a tan *δ* between 0.22 and 0.41 at 0.1 Hz are represented. (D) Products with a tan *δ* higher than 0.41 at 0.1 Hz are represented.

A total of 25 CFs were investigated in this study with a mean G′ value of 148.74 (98.9) Pa, 206.21 (106.4) Pa, 222.20 (107.1) Pa, 258.10 (108.0) Pa, and 270.80 (118.1) Pa tested at 0.1, 1.0, 1.6, 4, and 10 Hz, respectively. The calculated average change in G′ between 0.1 and 10 Hz was 122.06 (106) Pa [range: −21 to 367] representing an increase of 247.60% [range: 85–720] when compared to the values at 0.1 Hz. The respective increase in G″ was 333.43% [range: 56–699], whereas the increase in tan δ was 180.42% [range: 46–494], and 251.58% [range: 100–713] in G*. For more detailed data presentation see Tables 3 and 4 and Figures 2, 3, 4, 5.

**TABLE 3:** *G′*, *G″*, and tan *δ* values (in pascals) for the 25 measured cross‐linked soft tissue fillers at 0.1, 1.0, 1.6, 4, and 10 Hz. Values represent mean values for duplicate measurements.

	Mean *G′* (Hz)						Mean *G″* (Hz)						Mean tan *δ* (Hz)					
ID	0.1 Hz	1 Hz	1.6 Hz	4 Hz	10 Hz	Change (%)	0.1 Hz	1 Hz	1.6 Hz	4 Hz	10 Hz	Change (%)	0.1 Hz	1 Hz	1.6 Hz	4 Hz	10 Hz	Change (%)
BS2	43.7	98.5	119.1	173.3	282.7	546.9	27.8	67.5	78.9	101.6	127.1	357.2	0.64	0.69	0.66	0.59	0.45	−29.4
BS3	61.7	138.2	167.1	239.2	327.8	431.3	38.7	92.3	106.5	134.3	157.0	305.7	0.63	0.67	0.64	0.56	0.48	−23.6
BS4	185	315.7	353.3	442.7	552.1	198.4	82.0	131.9	144.0	169.8	197.0	140.2	0.44	0.42	0.41	0.38	0.36	−18.1
BS6	60.7	126.3	149.1	208.8	285.8	370.8	33.6	75.4	87.2	111.4	132.6	294.6	0.55	0.60	0.59	0.53	0.46	−16.3
BS7	143.5	241.7	271.9	341.7	421	193.4	61.9	104.9	114.0	131.3	148.7	140.2	0.43	0.43	0.42	0.38	0.35	−18.6
C1	187.6	243	252.5	269.5	284	51.4	48.7	39.2	36.9	34.9	36.9	−24.2	0.26	0.16	0.15	0.13	0.13	−50.0
C2	50.1	77.6	86.2	108.1	138.3	176.1	15.3	30.6	35.3	47.6	64.8	323.5	0.31	0.39	0.41	0.44	0.47	52.94
C3	99.6	134.3	145.3	172.9	210.5	111.4	21.7	40.6	46.6	61.8	82.7	281.1	0.22	0.30	0.32	0.36	0.39	77.3
C4	499.9	573.2	583.6	601.8	616.6	23.3	78.3	46.7	43.2	40.7	44.2	−43.6	0.16	0.08	0.07	0.07	0.07	−56.25
C5	28.8	77.8	96.1	149.3	207.4	620.1	22.0	57.8	69.1	97.9	153.8	599.1	0.76	0.74	0.72	0.66	0.74	−2.60
C6	79	153.8	176.8	238	297	276.0	39.4	78.0	89.7	117.7	154.1	291.1	0.50	0.51	0.51	0.50	0.52	4.01
C7	116.9	203.8	227.7	274.2	242.5	107.4	48.1	90.3	103.0	132.9	174.3	262.4	0.41	0.44	0.45	0.49	0.72	74.51
C8	175.4	229.5	244	277.9	321.6	83.4	39.0	55.8	61.6	76.5	97.2	149.2	0.22	0.24	0.25	0.28	0.30	36.04
C9	218.00	242.20	247.40	260.00	278.10	27.57	25.2	22.1	24.0	30.4	41.5	64.7	0.12	0.09	0.10	0.12	0.15	25.00
C10	127.8	147	152.6	166.6	184.3	44.2	17.8	23.6	27.0	36.4	50.6	184.3	0.14	0.16	0.18	0.22	0.27	92.85
C11	65.4	107.8	118.3	137.8	113.4	73.4	25.0	39.2	43.6	55.2	85.1	240.4	0.38	0.36	0.37	0.40	0.75	97.38
C12	165.7	221.8	236.5	263.5	199.7	20.52	34.3	61.3	70.0	93.4	149.4	335.9	0.21	0.28	0.30	0.35	0.75	257.14
C13	156.1	226.6	245.4	290	323.4	107.2	41.3	67.9	75.8	95.7	125.1	202.9	0.27	0.30	0.31	0.33	0.39	44.44
C14	279.9	329.1	339.6	352.9	259.2	−7.4	38.7	51.4	57.5	76.1	111.2	187.3	0.14	0.16	0.17	0.22	0.43	207.14
C15	81.2	105.1	111.6	135.1	203.3	150.4	16.5	20.4	22.7	30.2	45.2	173.9	0.20	0.19	0.20	0.22	0.22	9.85
C16	130.4	162.5	169.7	178.8	110.4	−15.3	22.1	32.7	37.3	50.7	74.2	235.7	0.17	0.20	0.22	0.28	0.67	294.11
C17	158.3	205.9	218	244.6	236.1	49.2	31.0	47.3	53.8	72.1	106.6	243.9	0.20	0.23	0.25	0.30	0.45	124.0
C18	216.6	277.1	293.4	318.1	201.7	−6.9	36.2	69.9	81.4	111.5	166.4	359.7	0.17	0.25	0.28	0.35	0.83	388.23
C19	248.2	311.5	328.9	357.6	262.4	5.7	38.2	73.4	85.5	117.1	165.4	333.0	0.15	0.24	0.26	0.33	0.63	320.0
C20	139.1	205.3	220.9	250.2	210.8	51.6	40.9	60.6	67.6	87.2	121.8	197.8	0.29	0.30	0.31	0.35	0.58	100–0

**TABLE 4:** G* δ values (in pascals) for the 25 measured cross‐linked soft tissue fillers at 0.1, 1.0, 1.6, 4, and 10 Hz. Values represent mean values for duplicate measurements.

	Mean G* (Hz)					
ID	0.1 Hz	1 Hz	1.6 Hz	4 Hz	10 Hz	Change (%)
BS2	51.8	119.4	142.8	200.9	309.9	498.26
BS3	72.8	166.2	198.2	274.3	363.4	399.18
BS4	202.3	342.2	381.5	474.1	586.2	189.77
BS6	69.4	147.1	172.7	236.6	315.0	353.89
BS7	156.3	263.4	294.8	366.1	446.5	185.67
C1	193.9	246.2	255.2	271.7	286.3	47.65
C2	52.4	83.4	93.2	118.1	152.7	191.41
C3	102.0	140.3	152.6	183.6	226.2	121.76
C4	506.0	575.1	585.2	603.2	618.2	22.17
C5	36.2	96.9	118.4	178.5	258.2	613.26
C6	88.2	172.5	198.3	265.5	334.6	279.37
C7	126.4	222.9	249.9	304.7	298.6	136.23
C8	179.6	236.2	251.6	288.2	335.9	87.03
C9	219.4	243.2	248.5	261.8	281.2	28.17
C10	129.0	148.9	154.9	170.6	191.1	48.14
C11	70.0	114.8	126.1	148.5	141.8	102.57
C12	169.2	230.2	246.7	279.6	249.4	47.40
C13	161.5	236.5	256.8	305.3	346.8	114.74
C14	282.6	333.1	344.4	361.0	282.1	−0.18
C15	82.8	107.0	113.9	138.4	208.2	151.45
C16	132.3	165.7	173.7	185.9	133.0	0.53
C17	161.3	211.2	224.6	255.1	259.1	60.63
C18	219.6	285.8	304.5	337.1	261.5	19.08
C19	251.1	320.0	339.8	376.3	310.2	23.54
C20	145.0	214.1	231.0	264.9	243.4	67.86

### 3.3 Comparison Between Cross‐Linked and Non‐Cross‐Linked Fillers

**FIGURE 6:**
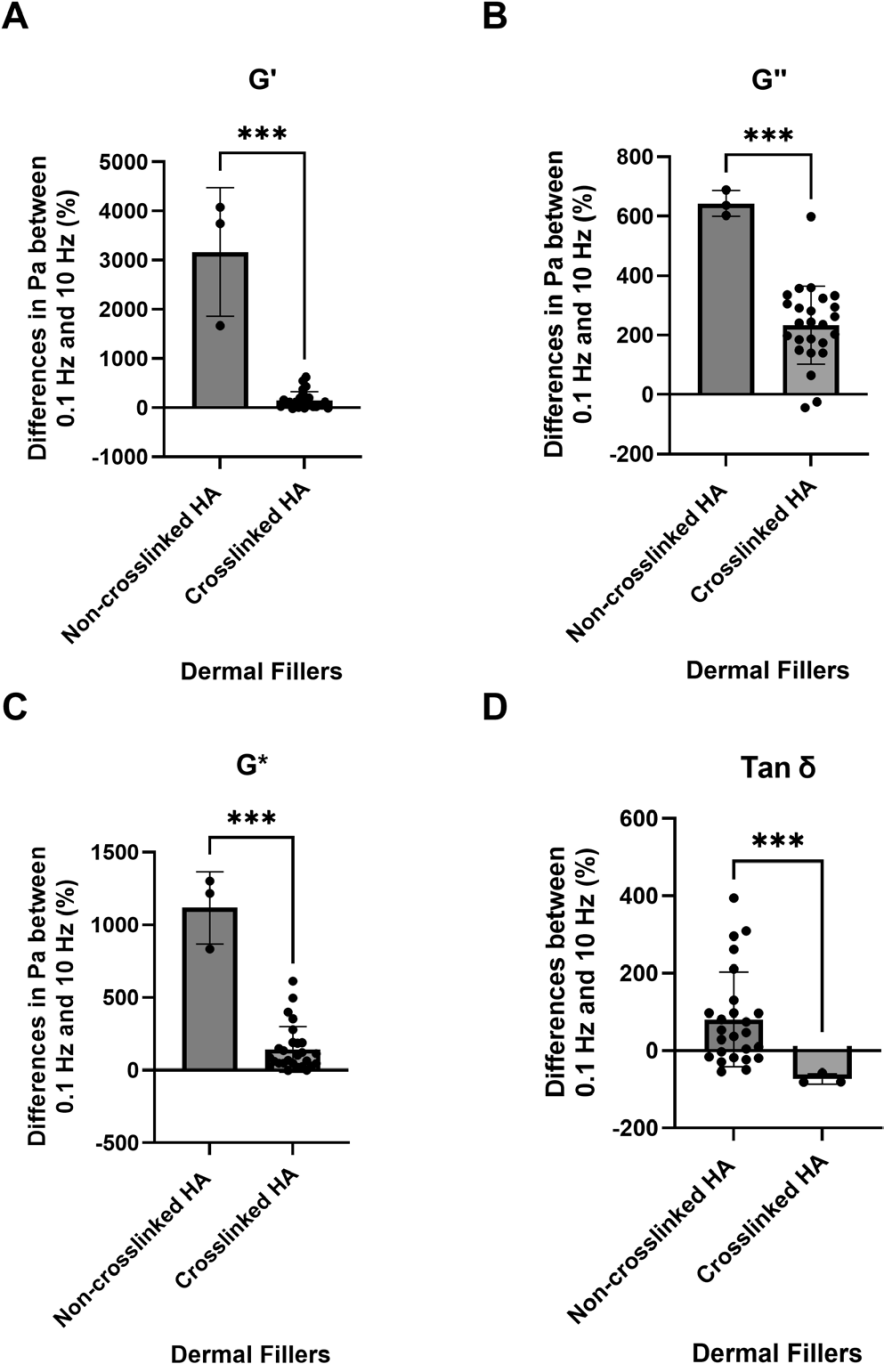
Comparison between changes in rheological values across between 0.1 and 10 Hz between non‐cross‐linked HA fillers and cross‐linked Ha fillers. (A) Elastic storage modulus (*G′*). (B) Viscous loss modulus (*G″*). (C) Complex viscosity modulus (G*). (D) tan *δ*. *** Significant differences (*p* < 0.001) according to the Mann–Whitney test.

The calculated difference between the change in percentage of CFs versus NCFs was 3016% (755) for G′ with *p* < 0.001 (CFs 95% CI: 75.67–219.5; NCFs 95% CI: −87.75 to 6409), whereas it was 926% (498) for G″ with *p* < 0.001 (CFs CI: 179.1–287.7; NCFs CI: 534.9–751.0), −154% (25.8) for tan δ with *p* < 0.001 (CFs 95% CI: −107.4 to −39.64; NCFs 95% CI: 29.76–131.1), and 966% (147) for G* with *p* < 0.001 (CFs 95% CI: 82.26–206.6; NCFs 95% CI: 500.6–1735) (Figure 6).

## 4 Discussion

This study investigated frequently used rheologic parameters (G′, G″, tan δ, and G*) of 28 commercially available HA‐based soft tissue fillers of which 3 were NCFs and 25 were CFs. The selected products represent the wide spectrum of currently used fillers in the esthetic market and were randomly selected without specifications for inclusion/exclusion for the purposes of this study (Table 1). Upon changing the testing frequency from 0.1 to 10 Hz, all 28 investigated fillers displayed changes in their viscoelastic properties (i.e., increase in G′, G″, tan δ, and G*values) with NCFs showing statistically significant greater increases when compared to CFs (Figure 6).

The experimental design of changing the testing frequency (from 0.1 to 10 Hz) was applied to simulate the varying degrees of stress that can act upon injected facial soft tissue fillers. Higher testing frequencies were used to simulate higher stress, which can be observed during dermal movements of various facial expressions and the resulting formation of dynamic and static rhytids. Lower testing frequencies simulate lower stress, which can be related to the limited movement of deep facial fat compartments during facial expressions like smiling and others [11].

The types of soft tissue fillers included in this study (NCFs and CFs) have two generally different characteristics which can be broken down to their particle size. Due to the cross‐linking process, the particle size is artificially increased by linking multiple HA molecules together and thereby increasing the relative percentual occupation of the particles within the gel. NCFs lack the cross‐linking process and therefore the relative particle size is smaller and the occupation of the particles within the gel is reduced when compared to CFs. Despite multiple other differences existing, which also vary between the different manufacturers, the above simplification may help to increase understanding and clinical applicability.

The results obtained in this study confirm previous investigations which have highlighted the non‐Newtonian behavior of HA‐based soft tissue fillers [8, 12, 13, 14]. This specific viscoelastic behavior is different from Newtonian fluids like water as their viscoelasticity increases due to the increase in shear forces as a result of the applied stress. When applying this knowledge to facial biomechanics, it may be hypothesized that HA‐based soft tissue fillers support the facial soft tissues to withstand mechanical stress by increasing their viscoelasticity and providing internal stability due to the increase in stiffness and viscosity of its gel. At the same time, the injected materials allow for continuous facial movements because they are gels and not solid implants.

The mechanical testing conducted in this study revealed however major differences between HA‐based soft tissue fillers with and without cross‐linking. NCFs displayed a statistically significant higher increase in all measured biophysical parameters (G′, G″ tan δ, and G*) when compared to CFs, with *p* < 0.001 in all cases (Figure 6). This increase suggests that both stiffness (solid behavior) and viscosity (fluid behavior) increase when stress is applied. This interesting behavior, which is different from CFs, predisposes us to implant NCFs in areas where high internal support for the facial soft tissues is desired. However, this hypothesis warrants further in vivo investigation.

Recent anatomic and ultrasound‐based investigations have revealed that the dermis and the more superficially located fascial planes move more than deeper planes. Additionally, it is known that the dermis is the location where rhytids are formed which undergo a slow transition from dynamic to static rhytids. These anatomic and biomechanical considerations may support the potential use of NCFs for dermal applications, particularly in facial regions with higher mobility. Nonetheless, clinical studies are needed to validate these in vitro‐based hypotheses. Due to their more stable viscoelastic behavior, CFs could be considered more suitable for deep product applications; however, this interpretation should be confirmed with clinical data. With a lower increase in viscoelastic properties, CFs are better suited for deep product application as they do not exhibit these large changes when exposed to stress. This would allow CFs to not inhibit natural facial mobility and to thereby “flow” with the various facial expressions intended. Current treatment algorithms mirror the results presented herein as CFs are mostly indicated for soft tissue volumization whereas NCFs are majorly indicated for dermal structural support.

Due to the administration of CFs and NCFs in different facial layers, the safety profile is different. A paucity of facial arteries can be found intradermally, whereas it is known that the majority of the 3‐dimensional facial arterial vasculature is located within the subcutaneous soft tissue.

However, due to the particle size difference between NCFs and CFs (i.e., smaller vs. larger, respectively), it can be deduced that NCFs are degraded faster than CFs, which influences treatment longevity and esthetic outcome duration. This requires a different treatment regime, which will influence patient handling and managing patient expectations.

Our findings confirm that NCFs and CFs possess fundamentally different viscoelastic properties, despite sometimes being marketed for similar clinical applications. Understanding these differences is essential for informed product selection and appropriate clinical use. Moreover, despite manufacturers suggesting similar uses for certain types of fillers (e.g., deep supraperiosteal applications), it is striking that even within the same category of fillers, the biophysical properties can differ significantly. This variability underscores the importance of thoroughly understanding the biophysical characteristics of each product, enabling injectors to tailor their choices according to anatomical layer, indication, and desired outcome. A deeper appreciation of these differences is essential to optimize treatment safety, performance, and longevity.

A strength of this study is the independent laboratory testing of all soft‐tissue fillers, ensuring unbiased and reliable results. This approach eliminates potential conflicts of interest and reduces bias, enhancing the credibility and scientific integrity of our findings. However, there are some limitations that must be acknowledged. Firstly, the market offers a wide variety of soft‐tissue fillers, and not all were included in this study. Only three NCFs were assessed—HYAcorp Fine, Genefill Fine, and Profhilo. These products were selected based on their availability in the market, relevance in clinical practice, and their differing HA concentrations (14 mg/mL for HYAcorp Fine and Genefill Fine, and 64 mg/mL for Profhilo), allowing us to explore performance differences among formulations. Nonetheless, the small number of products limits the generalizability of the results. Another limitation is the lack of consideration and evaluation of variability between different lots of the same product. Moreover, since all experiments were conducted in vitro, the study design inherently does not account for the effects of anatomy, shear stress, compression, or tissue integration on the fillers, which are crucial factors. This is significant, as it has been shown that rheological properties alone (e.g., G′) are not sufficient to fully understand a filler's clinical performance [15]. Future studies will need to elaborate on the findings presented herein and relate the results presented to in vivo clinical observations.

## 5 Conclusions

This study demonstrates that non‐crosslinked and crosslinked HA fillers exhibit fundamentally different rheological behaviors under shear stress. NCFs showed greater sensitivity to frequency changes, while CFs maintained more stable profiles. These differences highlight the need for clinicians to understand filler‐specific properties to optimize product selection and treatment outcomes. Future studies should aim to correlate these in vitro rheologic properties with in vivo performance and patient‐reported outcomes. Additionally, expanding the evaluation to include inter‐lot variability, degradation kinetics, and product behavior under dynamic anatomical conditions (e.g., facial movement, compression, and integration) would provide more comprehensive guidance for clinical practice.

## Author Contributions

S.F. and S.C. designed the study, analyzed the results, and wrote the manuscript. All the authors reviewed and approved the final version of the manuscript.

## Funding

This study was supported by BioScience GmbH, NA.

## Conflicts of Interest

S.F. is an employee of BioScience GmbH. The laboratory testing procedures were financially supported by BioScience, Germany. S.C. is the founder and CEO of Cotofana Anatomy a company specialized in anatomic education.

## Data Availability

The data that support the findings of this study are available on request from the corresponding author. The data are not publicly available due to privacy or ethical restrictions.
